# Editorial: Regeneration of plant organs *in vitro* and its mechanistic basis

**DOI:** 10.3389/fpls.2025.1568614

**Published:** 2025-03-05

**Authors:** Yun Li, Chenghao Li, Anwar Shahzad

**Affiliations:** ^1^ State Key Laboratory of Tree Genetics and Breeding, Engineering Technology Research Center of Black Locust of National Forestry and Grassland Administration, College of Biological Sciences and Technology, Beijing Forestry University, Beijing, China; ^2^ State Key Laboratory of Tree Genetics and Breeding, Northeast Forestry University, Harbin, China; ^3^ Department of Botany, Aligarh Muslim University, Aligarh, Uttar Pradesh, India

**Keywords:** plant regeneration, mechanistic basis, plant growth regulator, metabolites, light

Plant regeneration and the mechanisms involved are foundational to plant biology; investigating the development, differentiation, and regulatory mechanisms of plant organs can be done by utilizing *in vitro* culture techniques (Ikeuchi et al., 2016). In 1958, Steward (Steward et al., 1958) demonstrated that single cells derived from carrot phloem-induced callus tissue could regenerate into whole plants, while Skoog and Miller (Skoog and Miller, 1957) discovered that elevated auxin levels can stimulate root formation and elevated cytokinin levels promote shoot formation. These findings formed the basis of the totipotency theory of *in vitro* somatic plant cells. Leveraging this unique characteristic, researchers have developed methodologies to culture specific plant organs, such as leaf, root, stem, and flower, under controlled environmental conditions (Chen et al., 2016; Dai et al., 2022; Tymoszuk and Zalewska, 2014; Hosokawa et al., 1996). This methodology enables the investigation of internal and external factors, including nutrients, hormones, light, and temperature, which influence the formation and development of plant organs (Xu et al., 2023; Song et al., 2023). Furthermore, the exploration of signaling pathways, gene expression, and metabolic networks provided critical insights into the molecular basis of organ development. There are three primary plant regeneration pathways: tissue repair, *de novo* organogenesis, and somatic embryogenesis (Duclercq et al., 2011; Sugimoto et al., 2011). This research compiles innovative research articles and reviews that contribute to advancing the field.

There are various factors influencing plant regeneration, with plant growth regulators being among the most critical (Motte et al., 2014). Yan et al. optimized the culture conditions for each developmental stage of *Brettschneidera sinensis*; they found that the highest seed germination rate was achieved on Murashige and Skoog medium supplemented with 2.0 mg/L 6-benzylaminopurine (6-BA) and 0.2 mg/L 1-naphthaleneacetic acid (NAA). A combination of 1.0 mg/L 6-BA and 0.1 mg/L NAA promoted optimal shoot regeneration, while woody plant medium (WPM) was most effective for adventitious shoot elongation. Additionally, 1/2 MS medium containing 2.0 mg/L indole-3-butyric acid (IBA), 1.0 mg/L NAA, and 20 g/L sucrose resulted in the highest rooting rate. Molecular marker analyses using inter-simple sequence repeat (ISSR) and random amplified polymorphic DNA (RAPD) confirmed the genetic stability of regenerated plants. This study provides an effective technical framework for the conservation and propagation of *Brettschneidera sinensis*.

Brassinolide (BL), in addition to cytokinins and auxins, influence plant regeneration (Jia et al., 2019). Nie et al. investigated the effects of BL on somatic embryogenesis in *Pinus koraiensis* by applying varying concentrations of BL to callus tissues with different embryogenic potentials and assessing the physiological changes and hormone levels. Their findings demonstrated that callus tissues with different embryogenic capacities responded differently to BL. BL application stimulated the bioactivity of callus tissues, regulated cellular metabolism and hormone levels, reduced MDA (malondialdehyde) content, enhanced antioxidant enzyme activity, and influenced the phenylpropanoid metabolic pathway. Furthermore, the study identified the optimal BL concentrations for callus tissues with different embryogenic potentials, providing valuable insights for somatic embryogenesis in *Pinus koraiensis* and other conifers.

Metabolites play a critical role in plant regeneration (He et al., 2023). Chang et al. conducted a metabolomic and proteomic sequencing analyses on *Platycladus orientalis* cuttings from trees aged 5, 100, and 700 years. The results revealed that rooting rates and root numbers of cuttings from ancient trees were significantly lower than those from 5-year-old trees. Differentially accumulated metabolites (DAMs) in the phenylpropanoid and flavonoid biosynthesis pathways were more abundant in older trees, leading to lignification of the callus and inhibiting root formation. However, callus from 100-year-old cuttings showed significantly increased rooting rate. The wounding stimulated cell division and energy accumulation, while changes in associated metabolites facilitated the formation of adventitious roots. These findings offer a novel approach to addressing the challenges of propagating hard-to-root plants through cuttings.

The external environment plays a crucial role in regulating plant regeneration. Han et al. reviewed the effects of light on plant regeneration, emphasizing the significant and complex influences of light intensity, spectrum, and photoperiod. Light intensity requirements vary among plant species during *de novo* shoot organogenesis, with blue and red light promoting adventitious shoot regeneration. Photoperiod exerts its effects by influencing hormonal activity and photosynthesis. Appropriate light intensity and spectra (e.g., red light) enhance embryogenesis, while photoperiod also impacts embryo induction. Similarly, adventitious root regeneration is affected by light, with plant-specific variations in response. In summary, light regulates plant regeneration by modulating photoreceptor-mediated signal perception, hormone levels, metabolic pathways, and gene expression. By leveraging emerging technologies, further exploration of light-regulated plant regeneration processes will provide a new theoretical basis for optimizing regeneration techniques.

In conclusion, the studies compiled in this Research Topic provide a foundational basis for exploring plant regeneration and the key factors in molecular mechanisms that influence it.
